# Uptake of Clinical Decision Support Systems Among Health Care Professionals in Six European Countries and the United States: Cross-Sectional Survey Within the I-CARE4OLD Project

**DOI:** 10.2196/85071

**Published:** 2026-06-11

**Authors:** Collin JC Exmann, Anna-Maria Hiltunen, Ira Haavisto, Anna Salminen, Maikki Messo, Mikko Nuutinen, Mark Hoogendoorn, Wiebe Boorsma, Elizabeth P Howard, Vanja Pešić, Mor Alon, Federica Mammarella, Rosa Liperoti, Olena Švihnosová, Daniela Fialová, Natalia Drapała, Katarzyna Szczerbińska, Anja Declercq, Hein PJ van Hout, Johanna De Almeida Mello

**Affiliations:** 1 Department of General Practice Amsterdam UMC Amsterdam The Netherlands; 2 Aging and Later Life Research Program Amsterdam Public Health Research Institute Amsterdam The Netherlands; 3 Nordic Healthcare group Helsinki Finland; 4 Department of Social and Clinical Pharmacy Faculty of Pharmacy Charles University Prague Czech Republic; 5 Deparment of computer science Vrije Universiteit Amsterdam Amsterdam The Netherlands; 6 Hebrew senior life The Hinda and Arthur Marcus Institute for Aging Research Boston, MA United States; 7 Boston College Connell School of Nursing Boston, MA United States; 8 Boston College School of Social Work Chestnut Hill, MA United States; 9 Checker Software Solutions Profility Inc Haifa Israel; 10 Fondazione Policlinico Universitario Agostino Gemelli IRCCS Rome Italy; 11 Università Cattolica del Sacro Cuore Milan Italy; 12 Department of Internal Medicine and Geriatrics 1st Faculty of medicine Charles University Prague Czech Republic; 13 Laboratory for Research on Aging Society, Chair of Epidemiology and Preventive Medicine Medical Faculty Jagiellonian University Medical College Kraków Poland; 14 Department of Internal Medicine and Geriatrics Jagiellonian University Kraków Poland; 15 LUCAS, Center for Care Research and Consultancy KU Leuven Leuven Belgium; 16 Center for Sociological Research KU Leuven Leuven Belgium; 17 Department of Oral Health Sciences KU Leuven Leuven The Netherlands

**Keywords:** machine learning, decision support systems, innovation, home care, prediction models, long term care, older adults

## Abstract

**Background:**

The use of Clinical Decision Support Systems (CDSS), such as clinical decision rules, algorithms, or machine learning-based applications, has gained attention in recent years. However, their adoption and effectiveness may vary across different health care systems and settings. For a CDSS to be adopted, it must effectively address the practical issues encountered by professionals; however, little research has been done to identify these needs and requirements.

**Objective:**

This study aims to describe and compare the current use of various decision-support and prediction tools in long-term care for older people across health professionals from 6 European countries and the United States.

**Methods:**

This study analyzed survey data from a CDSS pilot study in a purposive sample of health professionals working with older adults with complex chronic conditions from six European countries and the United States. The survey included participants’ general background information, their current use of decision support tools, and their attitudes on the potential benefits of CDSS. About 20 participants were sampled per country. Closed responses were analyzed using correlation coefficients and regression models, while open-ended responses were clustered in a qualitative manner, categorizing each response.

**Results:**

A total of 151 professionals (mean age 45.5, SD 11.6 years, 71.5%, 108/151 female) participated in the pilot study. Most participants were physicians (85/151, 56.3%) or nurses (57/151, 37.7%). About 51% (78/151) of the participants reported using CDSS, while 22.4% (34/151) used predictive CDSS, showing important variation across samples from the seven countries. The regression model for comfort with technology showed a positive association for openness to new technologies (β=0.622; *P*<.001), although an inverse significant association was found for age (β=–0.022; *P*<.001). No significant associations were found for the actual use of CDSS. Participants reported using CDSS mainly for diagnostic purposes or for guideline implementation, not aimed at prognostic information. In contrast, examples of prognostic tools were most frequently mentioned by respondents as being valuable improvements to clinical practice.

**Conclusions:**

While some countries’ samples reported well-integrated digital health infrastructures and higher CDSS adoption rates, others still face challenges in implementing these. However, we found multiple examples of emerging tools, and at the same time, an important demand for predictive CDSS. Our findings highlight the need for improvement of current CDSS implementation and both development and implementation of particularly predictive CDSS.

## Introduction

Clinical Decision Support Systems (CDSS) are integrated software applications designed to support healthcare professionals in making clinical decisions [1,2]. Using patient data and analytical techniques, these systems generate alerts, offer diagnostic support, and deliver evidence-based recommendations intended to improve clinical outcomes. These tools are designed to inform clinicians’ decision-making about individual patients [3]. In the context of caring for older adults, particularly those with complex chronic conditions (CCC), the usefulness of CDSS becomes even more apparent. This population, marked by multiple interacting conditions and complex care needs, presents clinicians with significant challenges in balancing the benefits and risks associated with interventions [4]. This challenge is especially evident in community-based settings and long-term care facilities, where frontline care providers may have limited access to specialists or advanced diagnostic resources [5,6]. In such environments, smart decision support systems can facilitate complex evaluations. However, the advantages of CDSS use should outweigh the barriers experienced by professionals [7].

Existing CDSS differ in their underlying methods, the extent to which they provide general or patient-specific advice, and the principles on which they are based. Previous research has classified these systems as either knowledge-based or non–knowledge-based systems [2,8]. Knowledge-based systems include decision rules derived from expert knowledge and clinical consensus [8]. Examples of knowledge-based systems are clinical guidelines with decision trees or treatment protocols. They may or may not be digitally integrated into electronic health records (EHRs) for automatic evaluation or be used as benchmarking. Non–knowledge-based systems include clinical algorithms, as well as machine learning (ML) models which are trained to evaluate certain outcomes or risks based on a given set of predictors. Like knowledge-based systems, these systems provide clinicians with decision-supporting information, but in a data-driven manner rather than based on clinical reasoning. An example of such a system is the CHA2DS2-VASc score, which uses a simple scoring algorithm to calculate the risk of thromboembolic cerebrovascular events in patients with atrial fibrillation [9]. Beyond knowledge-based, these systems can be seen as having a more diagnostic (outcome in the present), or predictive aim (outcome in the future) [10].

The relatively recent implementation of EHRs has created an important potential for CDSS implementation [11,12]. In particular, the integration of such systems within the EHR could automatically generate recommendations in real-time and leverage state-of-the-art ML techniques for application in health care. Indeed, the use of non–knowledge-based CDSS, such as clinical algorithms or artificial intelligence (AI) applications, has gained support in recent years [13]. However, critiques have also emerged, highlighting gaps in how to appropriately interpret the provided results and questioning the extent to which the information can be fully trusted [14,15].

Against this background, the EU-Horizon 2020-funded project “Individualized CARE for OLDer persons with CCC at home and in nursing homes (I-CARE4OLD)” developed a clinical decision support tool – the iCARE tool. This tool aims to support clinicians in making decisions about care paths and treatments for home care and residential care settings [16]. The tool generates individualized prognostications derived from ML on the risk of adverse outcomes, changes in functional health, and impact of interventions. It is based on internationally standardized routine care data from the interRAI assessment instruments. Millions of interRAI assessment records from the participating countries were used, linked to registry data on mortality, hospitalization, medications, and health care use [16,17]. The prototype of the iCARE tool was tested during a pilot consisting of multiple phases. This study focuses on the reported current and beneficial use of various CDSS by participating health care professionals caring for older adults with CCC, as collected in the pre-pilot phase of the pilot [18]. The aim of this study is to describe and compare the use of clinical decision support tools and attitudes toward future CDSS development among health care professionals caring for older adults with CCC.

## Methods

### Overview

This research is part of the larger study iCARE4OLD funded by the European Union in the Horizon 2020 framework [16]. The study used a cross-sectional design using mixed closed and open-ended survey questions to explore the use and perception of CDSS among health care professionals. The protocol of the study was published recently [18]. Data collection took place simultaneously in seven countries: Belgium, the Czech Republic, Finland, Italy, the Netherlands, Poland, and the United States. The study was ethically approved in the participating countries and all health care professionals gave their written informed consent for participation. The protocol describes the different phases of the study: the pre-questionnaire phase, the patient evaluation phase in the iCARE tool, and the post-questionnaire phase. In this study, data from the prequestionnaire phase were used for the analysis.

### Participants and Eligibility

Participants were healthcare professionals involved in the care of older adults (aged 65 years or older) with CCC, either in long-term care facilities or primary care. Eligible participants were required to be qualified professionals capable of making or proposing therapeutic decisions (eg, physicians, nurses, and physiotherapists) and interpreting interRAI assessments. We used purposive sampling combined with snowball sampling of at least 20 participants per country to ensure sufficient observations for analysis. This sampling was performed by each country’s local study lead among purposively selected professionals. To ensure consistency across countries, the study leads held regular meetings reporting the recruitment and data collection procedures.

Local coordinators followed a standardized, documented procedure: site liaison and approval; targeted invitations with a translated information sheet; brief eligibility prescreening against the four inclusion criteria; scheduling of sessions; written informed consent; and administration of a uniform testing protocol (prequestionnaire, iCARE testing session, decision‑loop where applicable, postquestionnaires).

### Data Collection

Data collection started in November 2024 and ended in January 2025. Clinicians completed an online questionnaire created for the study, consisting of background questions (socio-demographic information, as well as information about education, profession or position, years of professional experience, etc), questions related to the use of interRAI instruments, attitudes toward new technology, and both closed and open-ended questions on the use of CDSS. The English version of the questionnaire can be found in Multimedia Appendix 1. Local study leads and their teams translated the surveys into the local languages**.** Using both closed and open-ended questions can result in triangulation, making the findings more valid and permitting a deeper understanding of the data [19,20].

### Data Validation and Cleaning

The data were validated to ensure data integrity. Validation procedures included verifying that each respondent ID had a single set of responses across all corresponding questionnaires. Timestamp checks were conducted to confirm consistency with respondents’ records. The dataset was systematically reviewed to identify and correct any erroneous values.

### Statistical Analysis

Quantitative data were analyzed using descriptive statistical methods, bivariate analysis, and regression models to explore associations. Pearson correlations were used for continuous data and Spearman correlations for Likert scales. A linear regression model was built for being comfortable with technology and a logistic model for the actual use of CDSS. Age was used as a continuous variable in the linear model and as dichotomous variable (age<50 or age≥50 years) in the logistic model. Only two professions were used in the models to allow for comparison (nurse and physician). The software STATA SE (version 18.5; StataCorp LLC) was used in the analysis.

All qualitative open-ended responses were first translated into English by the local teams, and then all answers were thematically analyzed by an experienced researcher and independently reviewed by another researcher to ensure consistency. Any discrepancies were discussed collaboratively until consensus was reached. In case of ambiguity or unclear answers, the researchers discussed the interpretation to reach a consensus on classification. If no reliable interpretation could be made, the response was noted as unclear and excluded from thematic coding.

### Response Clustering

We systematically clustered responses from the 3 open-ended questions. These questions were (1) use of decision support systems, (2) use of predictive decision support systems, and (3) decision support tools participants considered helpful. We classified the reported CDSS as either “knowledge based” or “non-knowledge based,” as “diagnostic” or “prognostic,” and lastly into consensus-based categories based on the thematic analysis [2,8]. For the classification into either diagnostic or prognostic, we followed previously proposed definitions [10]. Diagnostic refers to CDSS which classify the current health status or event, whereas in a predictive CDSS, the outcome is a future event. Where possible, we searched for additional information on the reported CDSS systems to complement available information. This way, the CDSS could be classified more reliably.

### Ethical Considerations

Ethical approvals were granted in each participating country before the start of the pilot. Italy: Comitato Etico Territoriale Lazio 3 (ID7068); the Netherlands: Amsterdam UMC Toetsingscommissie (2024.0527); Belgium: Commissie Medisch Ethiek van de Universitair Medische Ziekenhuizen KU Leuven (No. S69591); Czechia: Ethics Committee of the Faculty of Pharmacy, Univerzita Karlova, H2020-965341,); Poland: the Research Ethics Committee at the Jagiellonian University Medical College (118.0043.1.237.2024); the United States: Advarra Institutional Review Board (No. 965341); Finland: Well-being Services County of North Karelia (6970/13.00.01/2024, § 5/2024 and Kainuu Metti Järvikallio (6970/13.00.01/2024, § 23/2024); Central Uusimaa (KEUHDno-2024-3384, § 87); and Helsinki (HEL 2024-010604 *t* 13 02 01). All participants gave their written informed consent to participate in the study, and data were anonymized and further pseudomized to link assessments. Some countries offered financial compensation to participants as described in the protocol of the study [14].

## Results

### Overview

Table 1 shows the characteristics of the 151 participants from seven participating countries. Clinicians’ mean age varied from 38.4 (SD 9.7) years in the Dutch sample to 51.6 (SD 10.3) years in the sample from the United States. Participants from Finland and the United States were predominantly female (19/20, 95%), while the proportion of women in the overall sample was 71.5% (108/151). The proportion of participants from home care or long-term care services was similar across 6 countries (47-53%), with the exception of the Czech sample, with 28% (7/25) professionals from home care and 72% (18/25) from long-term care.

Amongst participants, physicians were the most prevalent (85/151, 56.3%), followed by nurses (57/151, 37.7%). Most respondents had a master’s educational level (99/151, 65.6%), followed by 15.2% (23/151) with a bachelor’s degree and 13.9% (21/151) with a PhD. Clinical experience was most extensive in participants from the United States (24.7 years) and Belgium (21.9 years), and relatively lower in those from the Netherlands (12 years). Similar results were found for the experience with complex care for older people, with an average of 23.1 (SD 14.9) years in the sample from the United States and 10.8 (SD 7.8) years in the Dutch sample.

Regarding users’ comfort with technology, the average score was 3.9 out of 5, with the score ranging from 1 (not comfortable at all) to 5 (very comfortable). Participants from Italy (4.3) and the Czech Republic (4.4) reported higher levels of comfort, while participants from the Netherlands reported the lowest (3.3). The average overall openness to integrating new technologies was high (4.2/5), with the highest levels for professionals from the United States (4.7), Italy (4.6), and Poland (4.5).

Table 2 shows the correlations between the most important determinants of CDSS use. A positive and significant correlation was found between being open to adopting new technologies and being comfortable with using technology in the overall sample (0.579; *P*<.001). This was also found for health care professionals from Belgium, the Czech Republic, Finland, and Italy, with very strong correlations in the samples from the Czech Republic (0.933; *P*<.001) and Finland (0.943; *P*<.001). All other correlations were not found to be significant in the full sample. In addition, for the participants from Belgium and the Czech Republic, there was a positive association between being open to technology and the use of CDSS. In the United States’ sample, the association between the number of years of clinical experience and the use of predictive CDSS was negative and significant (–0.464; *P*=.04).

**Table 1:** Characteristics of participants and their use of the Clinical Decision Support System.

Characteristics		All	Belgium	Czech Republic	Finland	Italy	the Netherlands	Poland	United States
Participants, n		151	20	25	20	20	26	20	20
Age (years), mean (SD)		45.5 (11.6)	48.7 (10.7)	43.0 (15.6)	45.0 (9.0)	48.5 (6.9)	38.4 (9.7)	46.5 (11.7)	51.6 (10.3)
Gender female, n (%)		108 (71.5)	15 (75)	13 (5)	19 (95)	9 (45)	16 (61.5)	17 (85)	19 (95)
Home care, n (%)		71 (47)	10 (50)	7 (28)	11 (55)	10 (50)	13 (50)	10 (50)	10 (50)
Long-term care, n (%)		80 (53)	10 (50)	18 (72)	9 (45)	10 (50)	13 (50)	10 (50)	10 (50)
**Position, n (%)**									
	Nurse	57 (37.7)	12 (60)	0 (0)	15 (75)	0 (0)	7 (26.9)	7 (35)	16 (80)
	Physician	85 (56.3)	5 (25)	25 (100)	2 (10)	20 (100)	19 (73.1)	12 (60)	2 (10)
	Other	9 (6)	3 (15)	0 (0)	3 (15)	0 (0)	0 (0)	1 (5)	2 (10)
**Degree, n (%)**									
	Basic	8 (5.3)	0 (0)	0 (0)	4 (20)	0 (0)	0 (0)	0 (0)	4 (20)
	Bachelor’s	23 (15.2)	2 (10)	0 (0)	13 (65)	0 (0)	3 (11.5)	0 (0)	5 (25)
	Master’s	99 (65.6)	13 (65)	25 (100)	3 (15)	19 (95)	19 (73.1)	13 (65)	7 (35)
	Doctor	21 (13.9)	5 (25)	0 (0)	0 (0)	1 (5)	4 (15.4)	7 (35)	4 (20)
Years in clinical practice, mean (SD)		18.2 (12)	21.9 (12.5)	19.3 (17)	15.7 (8.4)	18.2 (6.3)	12.0 (8.4)	17.3 (11.4)	24.7 (13)
Years of experience with geriatric complex care, mean (SD)		15.9 (10.9)	19.4 (11.5)	10.7 (9.7)	14.2 (9)	18.3 (6.6)	10.8 (7.8)	17.4 (10.8)	23.1 (14.9)
Working experience with interRAI, n (%)		72 (47.7)	14 (70)	1 (4)	20 (100)	20 (100)	6 (23.1)	2 (10)	9 (45)
Years with use of interRAI tools, mean (SD)		4.8 (7)	4.6 (6)	0.8 (1)	8 (6.2)	15.3 (7.8)	1.2 (3.2)	2.5 (5.4)	3.5 (6.2)
Comfortable with using technology^a^, mean (SD)		3.9 (1.1)	4.1 (0.8)	4.4 (1.2)	3.9 (1.3)	4.3 (0.9)	3.3 (1)	3.9 (0.8)	3.8 (1.5)
Open to adopting new technologies^b^, mean (SD)		4.2 (1)	4.1 (0.7)	4.2 (1.3)	4 (1.2)	4.6 (0.6)	3.5 (1)	4.5 (0.7)	4.7 (0.5)
Have used CDSS in work, n (%)		78 (51.7)	7 (35)	17 (68)	18 (90)	3 (15)	15 (57.7)	9 (45)	9 (45)
Have used predictions, n (%)		34 (22.5)	2 (10)	6 (24)	3 (15)	4 (20)	12 (46.2)	1 (5)	6 (30)

^a^1 (Not comfortable at all) to 5 (Very comfortable).

^b^1 (Not open at all) to 5 (Very open).

**Table 2:** Cross-sectional bivariate analysis for the use of Clinical Decision Support System (CDSS) among health care professionals overall and in the seven individual countries.

Variables	All (N=141)	Belgium (n=20)	Czech Republic (n=25)	Finland (n=20)	Italy (n=20)	the Netherlands (n=26)	Poland (n=20)	United States (n=20)
Years of clinical experience x use of CDSS	–0.049	0.633^a^	–0.218	–0.237	–0.286	0.025	–0.115	0.025
Years of clinical experience x use of predictions	–0.045	0.385	–0.084	0.119	0.085	0.249	–0.006	–0.464^b^
Open to adopting new technologies x comfortable with using technology	0.579^c^	0.613^a^	0.933^c^	0.943^c^	0.621^a^	0.257	0.291	0.018
Open to adopting new technologies x the use of CDSS	0.094	0.494^b^	0.399^b^	0.423	0.288	–0.160	0.225	0.032
Open to adopting new technologies x the use of predictions	–0.096	0.191	–0.032	0.118	–0.086	–0.265	0.171	0.023
Comfortable with using technology x the use of CDSS	0.137	0.345	0.394	0.359	0.380	0.187	0.118	–0.187
Comfortable with using technology x the use of predictions	0.049	0.186	–0.033	0.032	0	0.102	0.030	0.406

^a^*P*<.01.

^b^*P*<.05.

^c^*P*<.001.

### Use of CDSS

A total of 78 (51.3%) participants reported using any type of CDSS in their daily work, while 34 (22.4%) reported using specific predictive CDSS. The rates for CDSS use in daily work varied across countries’ samples ranging from 15% (3/20) in Italy, 35% (7/20) in Belgium, to the highest rate of 90% (18/20) in Finland. Regarding the use of CDSS for predictions, the lowest rates were found in the Polish (1/20, 5%) and Belgian (2/20, 10%) samples, while the highest rate was found in the Netherlands (12/26, 46.2%).

A total of 132 open-text answers were provided about the use of CDSS, and 49 answers to the question on predictive CDSS, of which 105 (79.5%) and 38 (77.6%) were analyzed, respectively. For the general CDSS question, all the exclusions stemmed from the answer being too unclear and therefore not being classifiable. Such answers, for instance, were not specific enough (eg, “I use multiple algorithms”) or did not clearly mention a CDSS (eg, “thromboembolism”, “standard procedures”). For the predictive CDSS question, answers that concerned nonpredictive CDSS were also excluded (eg, “CORNELL”, a diagnostic instrument).

**Figure 1:**
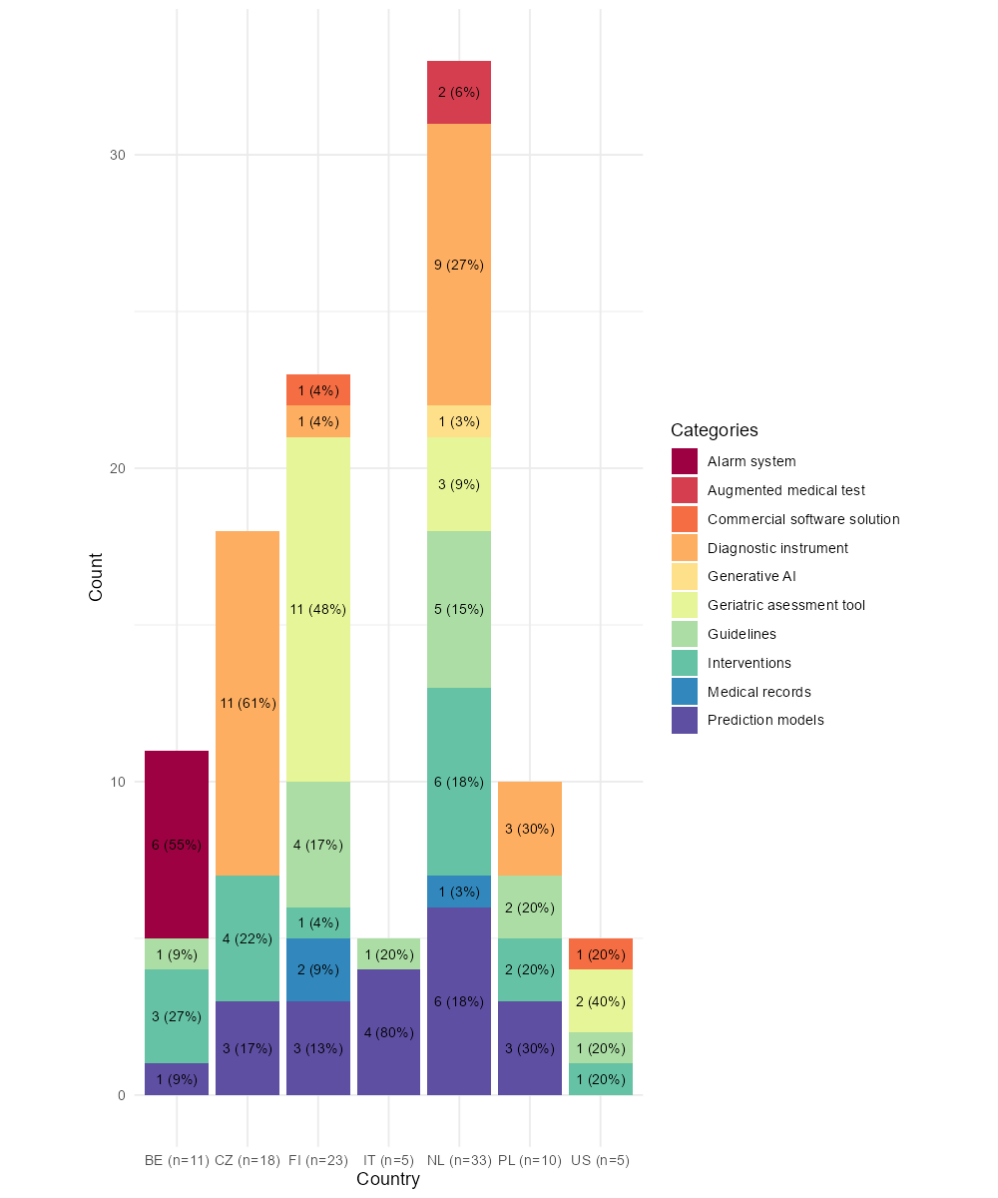
Use of any CDSS (question 1) among all health care professionals per country. CDSS: Clinical Decision Support System.

As can be seen in Figure 1, multiple dominant categories of CDSS were mentioned, such as diagnostic instruments (24/105, 23%), prediction tools (20/105, 19%), and tools considering intervention advice (17/105, 16%). Examples of these categories include the mini mental state examination or WELLS criteria (diagnostic instruments), CDSS on care pathways or future bleeding risks (prediction tools), and interaction checkers and antibiotic prescription systems (intervention advice). Another common category mentioned by professionals was geriatric assessment tools (16/105, 15%), most of which were considered interRAI instruments. Finally, some professionals mentioned commercial software solutions (N=2) providing one or more CDSS categories, but these responses lacked sufficient detail to identify specific categories or methods and were therefore grouped into a separate cluster.

When looking at the types of CDSS, most tools were knowledge-based (71/105, 68%) and aimed for diagnostic (69/105, 65%) purposes, thereby making predictive or prognostic tools much less common in current practice, only mentioned in about 19% (20/105) of the answers to the general CDSS question.

While analyzing the predictive CDSS mentioned, 24 (49%) answers had to be excluded, as they did not concern any predictive tool. Out of the remaining 26 valid predictive CDSS answers, most concerned either intervention advice (eg, CHA2DS2-VASc or CVRM treatment advice) or predictive tools such as (cardiovascular) mortality risk calculators. Some of the overall CDSS categories were excluded, as they are not predictive by definition. Note that in Figure 1, “prediction models” includes all CDSS providing direct outcome predictions based on responses to the general CDSS question.

### Regression Analysis for Comfortable Use and Current CDSS Use

Tables 3 and 4 show the results of the regression models built to explain being comfortable with using technology, and for the use of CDSS. In the first model, age and openness to new technologies were significant determinants of feeling comfortable with technology. Age showed an inverse association (β=–0.022; *P*<.001), while people who are more open to technology showed a significant positive association (0.622; *P*<.001). In the second model, no determinants were significantly associated with the actual use of CDSS.

**Table 3:** Results of linear regression for feeling comfortable using technology (N=141).

Characteristics	β coefficient (95% CI)	*P* value
Age	–0.022 (–0.035 to –0.009)	<.001^a^
Gender (ref: men)	–0.231 (–0.588 to 0.127)	.20
Profession (ref: nurse)	0.246 (–0.0827 to 0.575)	.14
Open to new technologies	0.622 (0.473-0.771)	<.001^a^

^a^*P*<.001. *R*^2^=0.427

**Table 4:** Results of the logistic regression model for the use of Clinical Decision Support System (N=141).

Characteristics	Odds ratios (95% CI)	*P* value
Age (ref: age<50)	0.766 (0.374-1.567)	.47
Gender (ref: men)	0.532 (0.232-1.222)	.14
Profession (ref: nurse)	1.888 (0.559-2.523)	.66
Open to new technologies	1.173 (0.447-3.077)	.75
Comfortable with technology	0.586 (0.255-1.343)	.21

### Future Beneficial CDSS

A total of 136 (90.1%) participants answered the question on what they perceive as future beneficial CDSS s in their clinical work (“What kind of clinical decision support tool would be beneficial for you in patient care?”), out of which we retrieved 179 valid answers, as some answers contained multiple technologies. These answers were qualitatively clustered in seven categories in total with individual subcodes as subcategories, as can be found in Table 5.

Frequently mentioned were systems that would either estimate risks and outcomes for patients, such as providing information on risk of mortality, hospitalization, or falls. Next, many professionals also mentioned that support systems giving advice on certain interventions (either pharmacological or nonpharmacological) could also be of help in their practice. Often, professionals would describe the need for personalized information on, for instance, risks for side effects or the effectiveness of the chosen intervention. These 2 categories, although depending on the exact use case, concern tools that would provide clinicians with prognostic information on treatment advice and important health outcomes. Especially, doctors seemed to be interested in such CDSS. Nurses were interested in these too, but more often reported future beneficial technology from the “other” category. We saw only a tendency toward more reporting of recommendation systems in the home care sample.

An important part of the answers also included the need for either electronic record adoption or improvements within an electronic record already in use. New functionalities such as automated note writing based on the recorded consultation or an integrated sharing between professionals were often mentioned. Finally, although generative AI was only mentioned once in the current CDSS use, multiple respondents answered that they would benefit from a clinical reasoning tool that would come up with differential diagnosis or with which they can discuss complex patient cases.

**Table 5:** Categories of future beneficial Clinical Decision Support System as reported by professionals in the study (iCARE4OLD pilot, cross-sectional study, November 2024-January 2025).

Category, N (% within column)			Total		Nurse		Doctor		Other		HC		LTCF
**Care pathways and needs**			8 (4)		4 (6)		2 (2)		2 (18)		5 (5)		3 (3)
	Care needs identification	4		—^a^		—		—		—		—	
	Care pathways software	2		—		—		—		—		—	
	Palliative care needs identification	1		—		—		—		—		—	
	Psychological care needs	1		—		—		—		—		—	
Clinical reasoning software			8 (4)		2 (3)		6 (6)		0 (0)		6 (6)		2 (2)
**Electronic record improvements**			19 (11)		11 (16)		8 (8)		0 (0)		10 (11)		9 (10)
	Automated record sharing	4		—		—		—		—		—	
	Automated record writing	7		—		—		—		—		—	
	Automated referral system	1		—		—		—		—		—	
	Electronic medical record summaries	4		—		—		—		—		—	
	Electronic medical record system	3		—		—		—		—		—	
**Other**			19 (11)		12 (18)		5 (5)		2 (18)		8 (9)		11 (13)
	interRAI	10		—		—		—		—		—	
	Alarm system	4		—		—		—		—		—	
	Other (single observations)	5		—		—		—		—		—	
**Protocol and guidelines**			6 (3)		4 (6)		2 (2)		0 (0)		0 (0)		6 (7)
	Communication protocol	1		—		—		—		—		—	
	Guideline improvements	4		—		—		—		—		—	
	Triage support	1		—		—		—		—		—	
**Recommendation systems**			67 (37)		19 (28)		46 (46)		2 (18)		41 (44)		26 (30)
	Diagnostic recommendation system	5		—		—		—		—		—	
	Interaction checker	15		—		—		—		—		—	
	Intervention recommendation system	15		—		—		—		—		—	
	Medication recommendation system	32		—		—		—		—		—	
Remote care and monitoring			6 (3)		6 (9)		0 (0)		0 (0)		2 (2)		4 (5)
**Risk and outcome predictions**			46 (26)		10 (15)		31 (31)		5 (45)		21 (23)		25 (29)
	Cancer	1		—		—		—		—		—	
	Delirium	3		—		—		—		—		—	
	Falls	5		—		—		—		—		—	
	Frailty	4		—		—		—		—		—	
	Health instability	3		—		—		—		—		—	
	Hospitalization	2		—		—		—		—		—	
	Mobility	1		—		—		—		—		—	
	Mortality and life expectancy	5		—		—		—		—		—	
	Pain	2		—		—		—		—		—	
	Physical restraints	1		—		—		—		—		—	
	Pressure ulcer	2		—		—		—		—		—	
	Rehabilitation outcomes	1		—		—		—		—		—	
	Thromboembolism	1		—		—		—		—		—	
	Unspecified	15		—		—		—		—		—	

^a^Not applicable.

## Discussion

### Principal Findings

This study provided an overview of the current use of CDSS by health professionals working with older persons with CCC from six European countries and the United States. The study highlighted notable variations in use rates and professionals’ needs for future developments in CDSS. The results also revealed differences in CDSS adoption. For example, adoption rates were as low as 15% (3/20) in the Italian sample, contrasted by remarkably high use at 90% (18/20) in the Finnish sample. These disparities are possibly influenced by varying degrees of investment in digital infrastructure, policy support mechanisms, and training, along with cultural attitudes to adopting technology in clinical practice [21,22]. In our study, most participants reported being open to new technology and comfortable using new technology, although the overall uptake of CDSS was 51.7% (78/151), and of predictive CDSS only 22.5% (34/151).

### Comparison With Previous Research

These results are similar to previous research indicating that professionals will adopt CDSS if they perceive benefits and are supported to implement the tools [23,24]. As a general principle, an effective CDSS must minimize the effort required by clinicians to receive and act on system recommendations [1]. However, as both a limitation of this study and a topic for reflection, we found a considerable proportion of unsatisfactory responses to questions regarding CDSS, particularly those addressing their predictive functionalities. This finding may indicate a lack of understanding among participants concerning the nature and capabilities of predictive CDSS. Prior studies have similarly identified a gap in healthcare professionals’ literacy on such topics, emphasizing the importance of addressing this issue to facilitate the effective implementation of CDSS in clinical practice [25-27].

Moreover, this study did not evaluate the performance of individual CDSS, which can be a topic of further research. Notably, certain reported CDSS (eg, generative AI) are not (yet) recommended for clinical practice. When combined with limited literacy among clinicians, the adoption of such tools could introduce risks to patient safety and care quality.

The findings from the study showed cross-national variations for comfort and openness to technology, and variation in the actual use of CDSS and predictions. Our findings showed that, although clinicians were comfortable or open to technology, this seldom correlated with the reported use, suggesting that although clinicians are keen to adopt such tools, there may remain infrastructural or systemic obstacles to real adoption. The results from the regression model for CDSS use are congruent with this. Previous research has also shown that differences in micro and macro-level factors across countries, for example, individual professionals’ views, costs, or national legal issues, can influence the adoption [28].

In addition, the results indicated that there is an important need among health care providers for improvements in CDSS. As most of our responses concerned either personalized risk assessments or general diagnostic or intervention recommendations, there is a practice-driven demand for further research improving healthcare prognostications on a personalized level [28]. This could, for instance, include personalized treatment advice [29]. This pilot was part of a project aiming at advancing the field on this subject, but as our data shows, there are many more outcomes or interventions to be studied.

Interestingly, consistent with previous research, we found a significant number of existing CDSS tools, even some of which could fill the needs mentioned as future beneficial CDSS, it seems CDSS are only used to a limited extent [30]. It has indeed been previously described that CDSS are often developed, but they often encounter challenges in achieving sustained implementation and long-term integration into practice [31-33]. This gap between development and real-world adoption highlights critical challenges in translating innovation into practice, such as integration with existing health IT systems, clinician training, workflow adaptation, and organizational readiness [34].

### Strengths and Limitations of This Study

We aimed to include participants with diverse paramedical backgrounds and from different countries, providing an overview across multiple countries and career stages. As only about 20 participants were included per country, the data cannot be regarded as representing the countries as a whole. Furthermore, the participant recruitment was, as described in the pilot protocol, based on targeted purposive samples and might therefore be biased toward research and innovation-oriented professionals and professionals with interRAI experience. However, it can be argued that for parts of the results, such as the future beneficial CDSS, we still reached a representative overview of what professionals could benefit from. Finally, as we worked with open-ended questions, the response categorization could still be influenced by subjective interpretations despite working with coding procedures. While we tried to retrieve additional information on the answered CDSS where possible, asking participants additional questions on the mentioned systems could have further refined the analysis. The responses to the questions also depend on the respondents’ understanding of the question. Although a trained facilitator was always available for any questions or clarifications, we found that not all answers fitted the original questions, mostly due to lack of knowledge or misinterpretation.

### Conclusions

This study collected the experiences with CDSS of healthcare professionals in a purposive sample across Europe and the United States. We found that CDSS use varied across countries samples, with a limited uptake in general, and particularly, for predictive CDSS. Participants mentioned multiple examples of CDSS they used, mostly for diagnostic rather than predictive purposes. Nevertheless, many professionals reported that predictive tools to inform them about patients’ health and intervention outcomes would be most beneficial. This highlights the need for further implementation of current systems and the development of more predictive CDSS.

## Data Availability

The datasets generated or analyzed during this study are available from the corresponding author on reasonable request.

## Appendix

Multimedia Appendix 1 Pre-questionnaire ICARE-tool pilot.

## Abbreviations

AI: artificial intelligence
CCC: complex chronic condition
CDSS: Clinical Decision Support System
EHR: electronic health record
I-CARE4OLD: Individualized CARE for OLDer persons with CCC at home and in nursing homes
ML: machine learning
