# Supplementary material for: Uptake of Clinical Decision Support Systems Among Health Care Professionals in Six European Countries and the United States: Cross-Sectional Survey Within the I-CARE4OLD Project
Source: J Med Internet Res. 2026 Jun 11;28:e85071. doi: 10.2196/85071 (PMC13305472; doi:10.2196/85071)
Supplement: Multimedia Appendix 1 [file jmir_v28i1e85071_app1.docx]

## ****Appendix 1****

Pre-questionnaire ICARE-tool pilot

| **Question** | **Answer option(s)** |
| --- | --- |
| **General information** |  |
| Age |  |
| Gender | Male  Female  NA |
| Current position/job |  |
| Clinical specialty (if applicable) |  |
| Role |  |
| Education/Degree |  |
| Workplace (nursing home / home care) |  |
| Number of years in clinical practice |  |
| Number of years of experience with older adults with complex chronic conditions |  |
| Any experience with interRAI tools? | Advanced level of proficiency  Some working experience  I have heard about interrail  No experience |
| Experience with use of interRAI tools (years) |  |
| **Attitudes towards new technology** |  |
| How comfortable are you with using technology in general in your daily professional activities? | 1-5 Likert scale (Not comfortable at all – Very comfortable) |
| How open are you to adopting new technologies in general in your healthcare practice? | 1-5 Likert scale (Not open at all – Very open) |
| What kind of clinical decision support tool would be beneficial for you in patient care? (Briefly describe) |  |
| Have you used any decision support systems in your work? | Yes/No  If yes, please state what kind of tool |
| Have you used any decision support systems with predictions for patient trajectories? If yes, what? | Yes/No  If yes, please state what kind of tool |
